# Aneurysmal Subarachnoid Hemorrhage in Pediatric DADA2: A Case Report and Literature Review

**DOI:** 10.7759/cureus.106332

**Published:** 2026-04-02

**Authors:** Philipp Becker, Tomas Dobrocky, Jan Gralla, Volker Umlauf, Julia Lehner, Deborah Bartholdi, Katharina Lutz, Klaus Tenbrock, Philippe Schucht, Philipe Breiding

**Affiliations:** 1 Institute of Diagnostic and Interventional Neuroradiology, Inselspital, Bern University Hospital, University of Bern, Bern, CHE; 2 Division of Paediatric Intensive Care Medicine, Department of Paediatrics, Inselspital, Bern University Hospital, University of Bern, Bern, CHE; 3 Division of Neuropaediatrics, Development and Rehabilitation, Department of Paediatrics, Inselspital, Bern University Hospital, University of Bern, Bern, CHE; 4 Department of Human Genetics, Inselspital, Bern University Hospital, University of Bern, Bern, CHE; 5 Department of Neurosurgery, Inselspital, Bern University Hospital, University of Bern, Bern, CHE; 6 Division of Paediatric Rheumatology, Department of Paediatrics, Inselspital, Bern University Hospital, University of Bern, Bern, CHE

**Keywords:** aneurysm, dsa, pediatric, stroke, vasculitis

## Abstract

Deficiency of adenosine deaminase 2 (DADA2) is a rare autosomal recessive autoinflammatory vasculopathy frequently associated with early-onset central nervous system involvement. Intracranial aneurysm formation and aneurysmal subarachnoid hemorrhage (aSAH) represent uncommon manifestations and remain poorly characterized from a neuroradiological perspective. We report the case of a nine-year-old boy with genetically confirmed DADA2 presenting with aneurysmal subarachnoid hemorrhage who underwent multimodal neurovascular imaging, including digital subtraction angiography (DSA). To contextualize this finding, a structured literature review was performed to identify previously reported cases of intracranial aneurysms in patients with genetically confirmed DADA2. Clinical presentation, hemorrhagic phenotype, aneurysm location, and treatment strategies were analyzed. In addition to our case, seven published patients with genetically confirmed DADA2 and intracranial aneurysms were identified, yielding a total of eight analyzed patients. Hemorrhagic events occurred in five of the eight patients, whereas three of the eight patients presented exclusively with ischemic manifestations. Aneurysms were predominantly multiple and located in the posterior circulation or distal vessels. Anti-TNF-α therapy was administered in seven patients, while aneurysm-directed intervention was reported in one case. These findings suggest that DADA2-associated aneurysms represent a distinct inflammatory neurovascular phenotype characterized by multiplicity and peripheral distribution. In pediatric patients presenting with unexplained aneurysmal subarachnoid hemorrhage, particularly in the context of systemic inflammatory features, DADA2 should be considered as a potential underlying etiology. Comprehensive vascular assessment with DSA may be essential for detecting small or atypically located aneurysms in this setting.

## Introduction

Deficiency of adenosine deaminase 2 (DADA2) is an autosomal recessive monogenic autoinflammatory disorder, first described in 2014, caused by loss-of-function mutations in the ADA2 (formerly CECR1) gene, with an estimated prevalence of approximately one in 222,000 individuals [1-3]. ADA2 encodes a dimeric extracellular enzyme predominantly secreted by myeloid cells that catalyzes the conversion of adenosine to inosine and plays a key role in immune regulation and vascular integrity [2,4,5]. Impaired ADA2 activity results in a systemic vasculopathy characterized by inflammation of small- and medium-sized arteries, with histopathological findings including periarteritis, fibrinoid necrosis of the media, and disruption of the internal elastic lamina [1,5,6]. The disease typically manifests in childhood and may involve multiple organ systems, reflecting primary inflammatory arteriopathy affecting small- and medium-sized vessels [1,5,7].

Clinically, DADA2 exhibits a broad phenotypic spectrum that can be categorized into three major domains: inflammatory/vascular, immune dysregulatory, and hematologic manifestations. The inflammatory phenotype is characterized by systemic vasculitis and early-onset cerebrovascular events, whereas immune dysregulation may manifest as hypogammaglobulinemia, impaired humoral responses, and lymphoproliferation. Hematologic involvement includes cytopenias, pure red cell aplasia, and, in severe cases, bone marrow failure. Importantly, these phenotypic categories are not mutually exclusive, and most patients demonstrate overlapping features across multiple domains, reflecting the multisystem nature of the disease [8]. This phenotypic heterogeneity reflects the underlying interplay between immune dysregulation and vascular injury.

At a mechanistic level, the vascular manifestations of DADA2 are thought to arise from dysregulated innate immunity with monocyte/macrophage-driven inflammation. ADA2 deficiency promotes polarization toward a proinflammatory M1 phenotype, leading to increased production of cytokines such as tumor necrosis factor (TNF), which appears to represent a central effector of vascular injury [5,8]. In parallel, experimental data suggest impaired endothelial integrity and direct disruption of endothelial cell layers by ADA2-deficient monocytes, supporting a model of combined immune-mediated vascular damage and intrinsic endothelial dysfunction [5]. Although the precise molecular function of ADA2 remains to be fully elucidated, these mechanisms provide a plausible explanation for the development of a systemic vasculopathy characterized by vessel wall damage, luminal alterations, and consequent structural vessel wall weakening predisposing to aneurysm formation.

Central nervous system involvement is one of the most clinically relevant and potentially severe manifestations of DADA2 and has been reported in up to half of affected patients [5,7]. Neurological events typically occur early in the disease course, often during childhood, and may constitute the initial presentation [2]. Imaging findings most commonly include recurrent lacunar ischemic infarcts involving the deep brain nuclei and brainstem, as well as intracerebral and subarachnoid hemorrhages (SAH) [4,7,9]. These manifestations are thought to reflect an underlying small- and medium-sized vessel vasculopathy, which may remain angiographically occult on routine imaging, posing a significant diagnostic challenge [7]. Importantly, in pediatric patients, hemorrhagic events such as subarachnoid hemorrhage are rare but clinically critical and may be associated with diagnostic delay, as small or peripherally located aneurysms may remain occult on initial cross-sectional imaging.

Intracranial aneurysm formation represents a rare but clinically significant neurovascular complication of DADA2. In contrast to sporadic intracranial aneurysms [7,9], DADA2-associated aneurysms often exhibit atypical features, including multiplicity, peripheral or non-branching locations, fusiform morphology, small size at rupture, and a predilection for posterior circulation and spinal arteries [6,7]. These characteristics suggest that wall fragility stems from endothelial dysfunction and transmural inflammation, rather than degenerative changes [5,6]. Consequently, aneurysmal subarachnoid hemorrhage (aSAH) in DADA2 poses a significant diagnostic challenge in pediatric patients, and small aneurysms may elude detection on conventional imaging or the presence of multiple vascular lesions may make it impossible to definitively identify the specific rupture site [7,9].

The diagnosis of DADA2-associated neurovascular complications is challenging because of the predominant involvement of small- and medium-sized vessels, which are frequently beyond the resolution of cross-sectional imaging techniques [4,7]. Digital subtraction angiography (DSA) remains the reference standard for comprehensive vascular assessment in this setting [4,6,7].

The purpose of this report was to describe a rare case of aSAH in a pediatric patient with DADA2 and highlight its characteristic neurovascular imaging features. By doing so, we aimed to raise awareness of this uncommon complication and emphasize the role of advanced vascular imaging in the diagnostic workup of unexplained pediatric SAH.

## Case presentation

Patient history and clinical background

**Figure 1:**
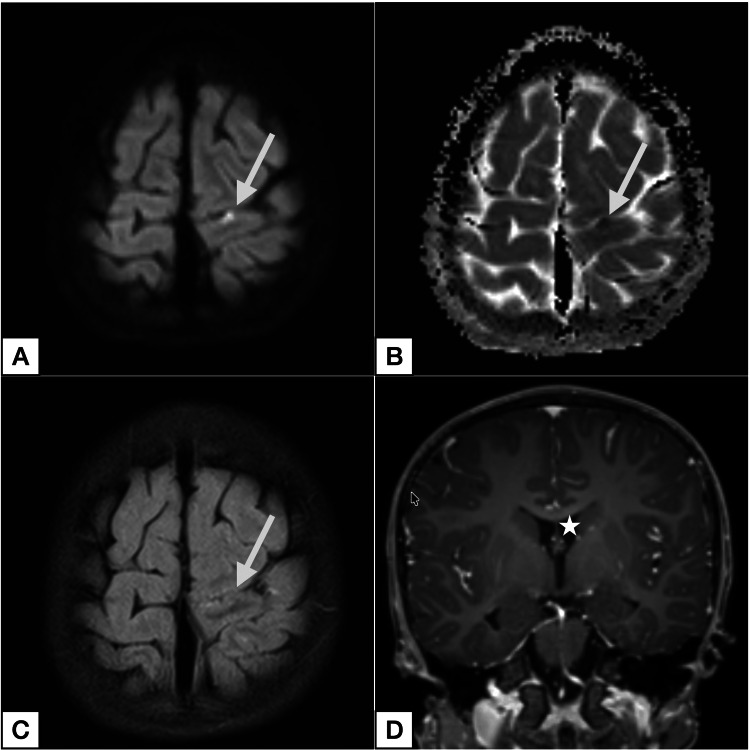
Magnetic resonance imaging at initial ischemic presentation (nine months prior to aneurysmal subarachnoid hemorrhage) (A) Axial diffusion-weighted imaging demonstrating focal cortical diffusion restriction in the left precentral gyrus (arrow). (B) Corresponding apparent diffusion coefficient map (arrow) confirming true restriction with matching hypointensity. (C) Axial fluid-attenuated inversion recovery sequence without clear lesion demarcation (arrow), consistent with acute ischemia. (D) Coronal contrast-enhanced T1-weighted imaging demonstrating normal ventricular size (asterisk).

A nine-year-old boy with a known diagnosis of DADA2-associated vasculopathy was admitted to our institution. Diagnosis was established based on clinical presentation and genetic confirmation. At the time of presentation, the patient was administered immunomodulatory treatment with canakinumab. His medical history was notable for a previous brain ischemia (Figure 1), which had occurred nine months prior to the hemorrhage. The initial clinical presentation included weakness of the right arm. At that time, magnetic resonance angiography (MRA) showed no evidence of intracranial aneurysmal disease. There was no history of trauma, coagulation disorders, or infectious endocarditis.

Acute presentation

The patient presented with sudden onset of severe headache, accompanied by nausea and repeated vomiting. Upon arrival of emergency medical services, the Glasgow Coma Scale (GCS) score was 15 with stable airway, breathing, and circulation. During the prehospital course, progressive neurological deterioration occurred with increasing somnolence and repeated vomiting. Shortly after arrival in the emergency department, the neurological status deteriorated further. The patient became soporous with a GCS score <8, accompanied by bradypnea, bradycardia (50 beats/min), and severe arterial hypertension up to 250/130 mmHg. Pupils were isocoric and promptly reactive to light. Emergency endotracheal intubation was performed. During induction of anesthesia, a possible seizure event was observed, characterized by extensor posturing of the arms and upward gaze deviation. Following stabilization measures, including head elevation and hyperosmolar therapy with 3% sodium chloride, urgent CT-based neuroimaging was obtained.

**Figure 2:**
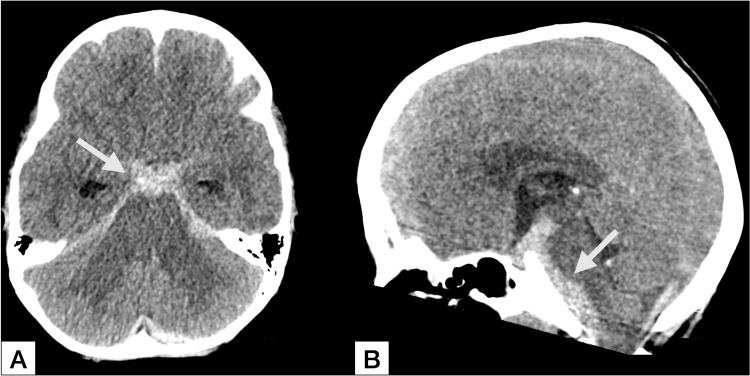
Initial non-contrast computed tomography imaging Axial (A) and sagittal (B) non-contrast head computed tomography imaging demonstrating diffuse subarachnoid hemorrhage, with predominant involvement of the basal cisterns in (A) and the posterior fossa in (B) (arrows).

Initial non-contrast head CT (Figure 2) demonstrated diffuse SAH, predominantly involving the basal cisterns and posterior fossa, consistent with Fisher grade 4, reflecting a high subarachnoid blood burden with associated hydrocephalus. No intraparenchymal hematoma was identified. Subsequent CT angiography showed no evidence of vascular bleeding. After imaging, an external ventricular drain was then placed for acute hydrocephalus (opening intracranial pressure 18 mmHg), and blood pressure was managed to maintain adequate cerebral perfusion. Coagulation was optimized with intravenous prothrombin complex concentrate (300 IU) and vitamin K (10 mg) in response to an international normalised ratio (INR) of 1.27.

Initial management included airway protection, external ventricular drainage for hydrocephalus, blood pressure control, and hyperosmolar therapy. Despite these measures, no immediate neurological improvement was observed, and the patient remained in a critical condition with impaired consciousness and signs of increased intracranial pressure, necessitating intensive care management. On admission to the ICU, the neurological status corresponded to World Federation of Neurosurgical Societies (WFNS) grade 4.

**Figure 3:**
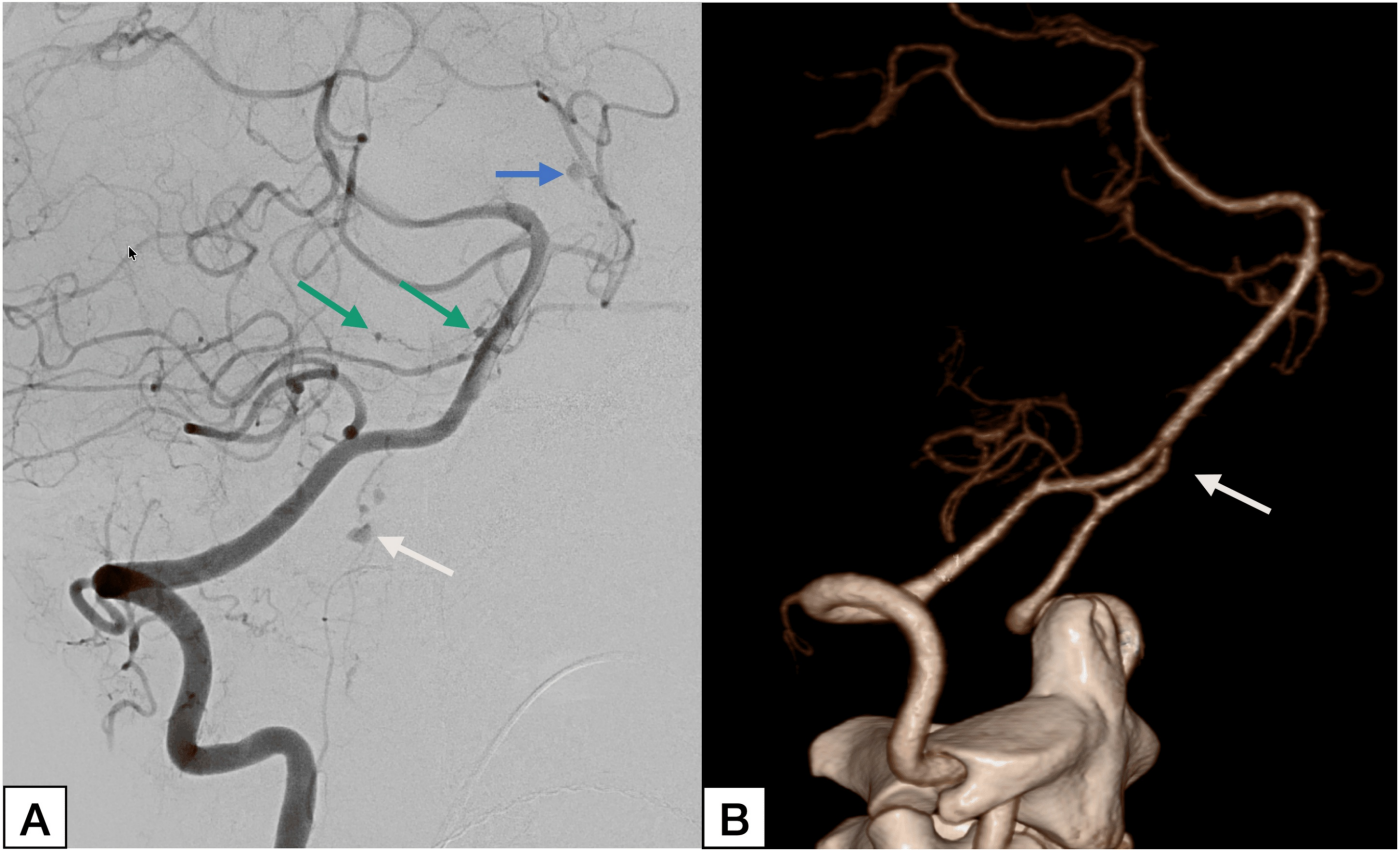
Detection of small DADA2-associated intracranial aneurysms using digital subtraction angiography versus computed tomography angiography (A) Oblique right vertebral artery digital subtraction angiography demonstrating multiple small aneurysms of basilar perforator arteries (green arrows), the left superior cerebellar artery (blue arrow), and the superior segment of the anterior spinal artery (white arrow). The multiplicity, small size, and involvement of distal posterior circulation and spinal vessels are atypical for sporadic saccular aneurysms and suggest an underlying inflammatory arteriopathy. (B) Three-dimensional reconstruction of computed tomography angiography of the same vascular territory demonstrating no detectable aneurysms; the anterior spinal artery aneurysm (white arrow) is not visualized.

Given the inconclusive findings on CT angiography and the extent of SAH, DSA was performed under general anesthesia (Figure 3). Selective angiographies of the internal carotid, external carotid, and vertebral arteries were obtained bilaterally. The study revealed multiple small intracranial aneurysms of the posterior circulation involving the V4 segment of the vertebral artery, the left posterior inferior cerebellar artery, the left superior cerebellar artery, bilateral anterior inferior cerebellar arteries, and the perforator branches of the basilar artery. Additionally, five aneurysms were identified along the superior segment of the anterior spinal artery. An aneurysm of the anterior spinal artery was suspected as the potential rupture site, based on contrast pooling and signs of incipient thrombosis. However, due to the multiplicity of aneurysms, a definitive bleeding source could not be established.

Diagnostic considerations

The identification of multiple small, atypical aneurysms within the posterior circulation and anterior spinal artery, occurring in the context of established DADA2, was highly suggestive of DADA2-associated inflammatory arteriopathy. Aneurysm morphology, peripheral distribution, and absence of a dominant saccular lesion were considered atypical for sporadic intracranial aneurysms. Infectious, traumatic, and atherosclerotic etiologies were deemed unlikely based on the clinical history, laboratory findings, and imaging characteristics.

Therapeutic decision-making

The multiplicity of the aneurysms, their peripheral distribution, and the involvement of eloquent vascular territories rendered them unamenable to surgical or endovascular management without significant risk. Interventions targeted at the anterior spinal artery aneurysm (the presumed source of hemorrhage) posed a significant risk of iatrogenic spinal cord ischemia and associated severe neurological deficits. Therefore, management was conservative and guided by a multidisciplinary team involving neuroradiology, neurosurgery, pediatric neurology, pediatric rheumatology, and pediatric intensive care specialists. Vasospasm prophylaxis with a calcium channel blocker (nimodipine 60 mg orally six times daily) was administered and immunomodulatory therapy was adapted in the context of the underlying DADA2-associated vasculopathy, with subsequent initiation of anti-tumor necrosis factor (anti-TNF-α) therapy using etanercept (50 mg intravenously once weekly). Endovascular spasmolysis was not pursued, as the risk of iatrogenic hemorrhage in the context of fragile, inflamed vessels associated with DADA2 was considered to outweigh the potential benefit.

Clinical course and outcome

**Figure 4:**
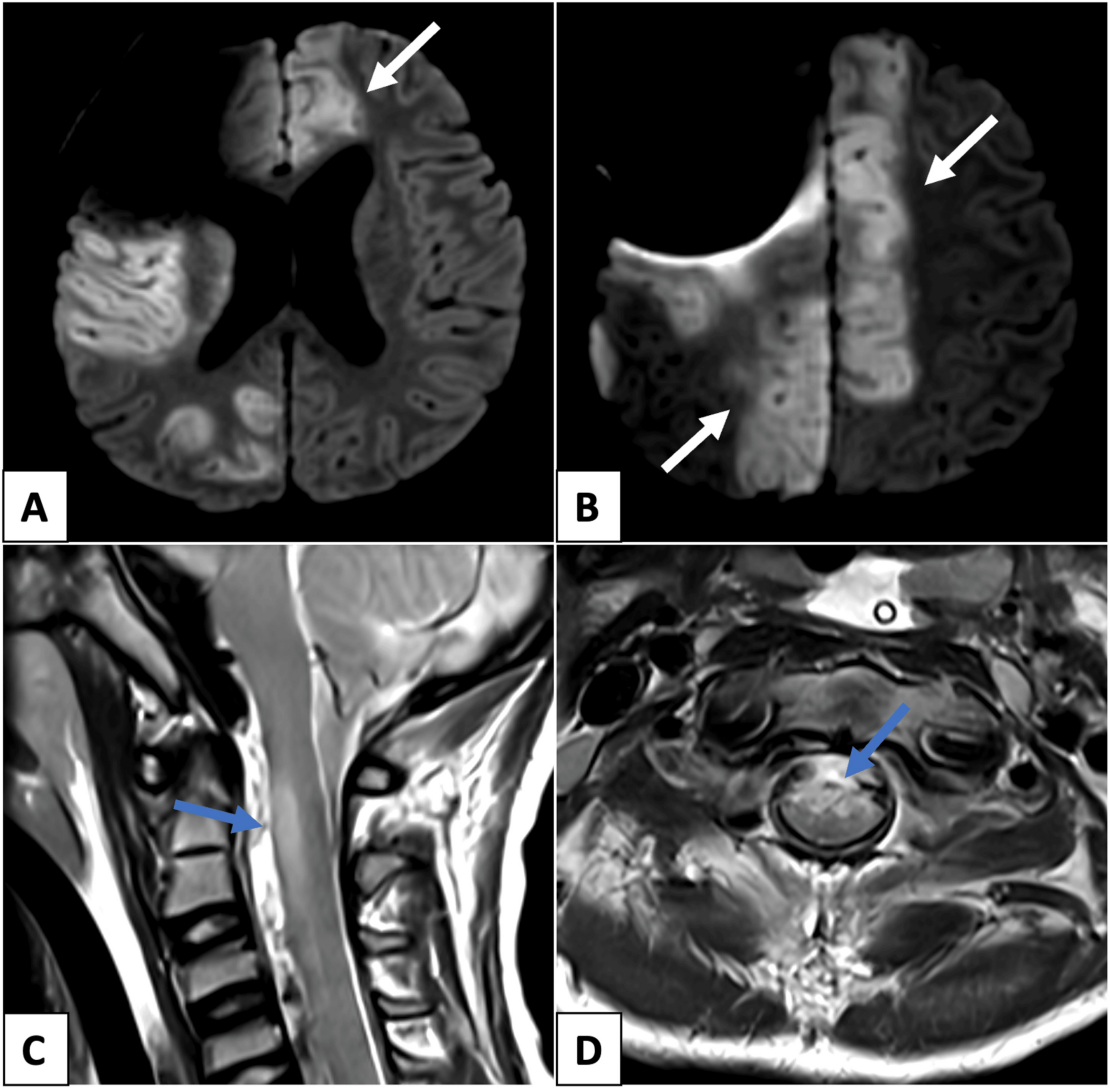
Magnetic resonance imaging of the brain at follow-up Axial brain diffusion-weighted imaging (A, B) shows extensive bilateral cortical ischemia involving the parasagittal regions (white arrow), accompanied by ventricular enlargement in (A). Sagittal (C) and axial (D) T2-weighted spine imaging reveal additional anterior cervical spinal cord ischemia (blue arrow).

The patient required prolonged intensive care owing to severe neurological injury with extensive cerebral and spinal ischemia (Figure 4). Early in the clinical course, spinal cord ischemia occurred, clinically manifesting with loss of deep tendon reflexes and subsequent absence of response to peripheral painful stimuli, most likely due to progressive thrombosis of the anterior spinal artery related to aneurysm-associated flow deceleration. During the subsequent vasospasm phase, severe vasospasms resulted in extensive subacute ischemic lesions involving the bilateral anterior cerebral artery territories and the right middle cerebral artery territory, as well as small multifocal ischemic lesions in the bilateral hippocampi and the splenium of the corpus callosum. The course was further complicated by recurrent intracranial pressure elevations exceeding 40 mmHg. Weaning from the external ventricular drainage was unsuccessful; therefore, a ventriculoperitoneal shunt was implanted on day 20. Following shunt placement, a basal ganglia hemorrhage occurred, not located along the catheter tract, and was therefore considered unlikely to be procedure-related; perioperative cardiovascular instability was considered the most likely contributing factor. Owing to the anticipated long-term ventilatory dependence related to the extent of central and spinal nervous system injury, surgical tracheostomy was performed. Follow-up neuroimaging demonstrated stable ventricular size without hemorrhage progression. After 14 weeks of intensive care, the patient was transferred to a neurorehabilitation facility.

In the following months, the patient experienced several episodes of transient decreased vigilance. Repeated cranial CT scans showed no new ischemic lesions or hemorrhage progression. Neurologically, he remained stable (GCS 15) with persistent left-sided hemiparesis; he was wheelchair dependent but able to stand with assistance.

Clinical timeline

The clinical course can be summarized as a biphasic neurovascular disease pattern in the context of genetically confirmed DADA2. Initially, the patient presented with ischemic cerebral events without evidence of intracranial aneurysms on MRI. The acute event marked a transition to a hemorrhagic manifestation, with sudden onset of severe headache and rapid neurological deterioration due to aneurysmal subarachnoid hemorrhage. Key red flags suggestive of an underlying DADA2-associated vasculopathy rather than sporadic aneurysmal disease included: (i) early disease onset, (ii) prior ischemic strokes, and (iii) the presence of multiple small, atypically located aneurysms involving the posterior circulation and anterior spinal artery.

## Discussion

Literature review

The institutional review board waived the requirement for ethical approval because this study involved the analysis of previously published data. The literature review was guided by the Preferred Reporting Items for Systematic Reviews and Meta-Analyses (PRISMA) 2020 guidelines [10]. 

**Figure 5:**
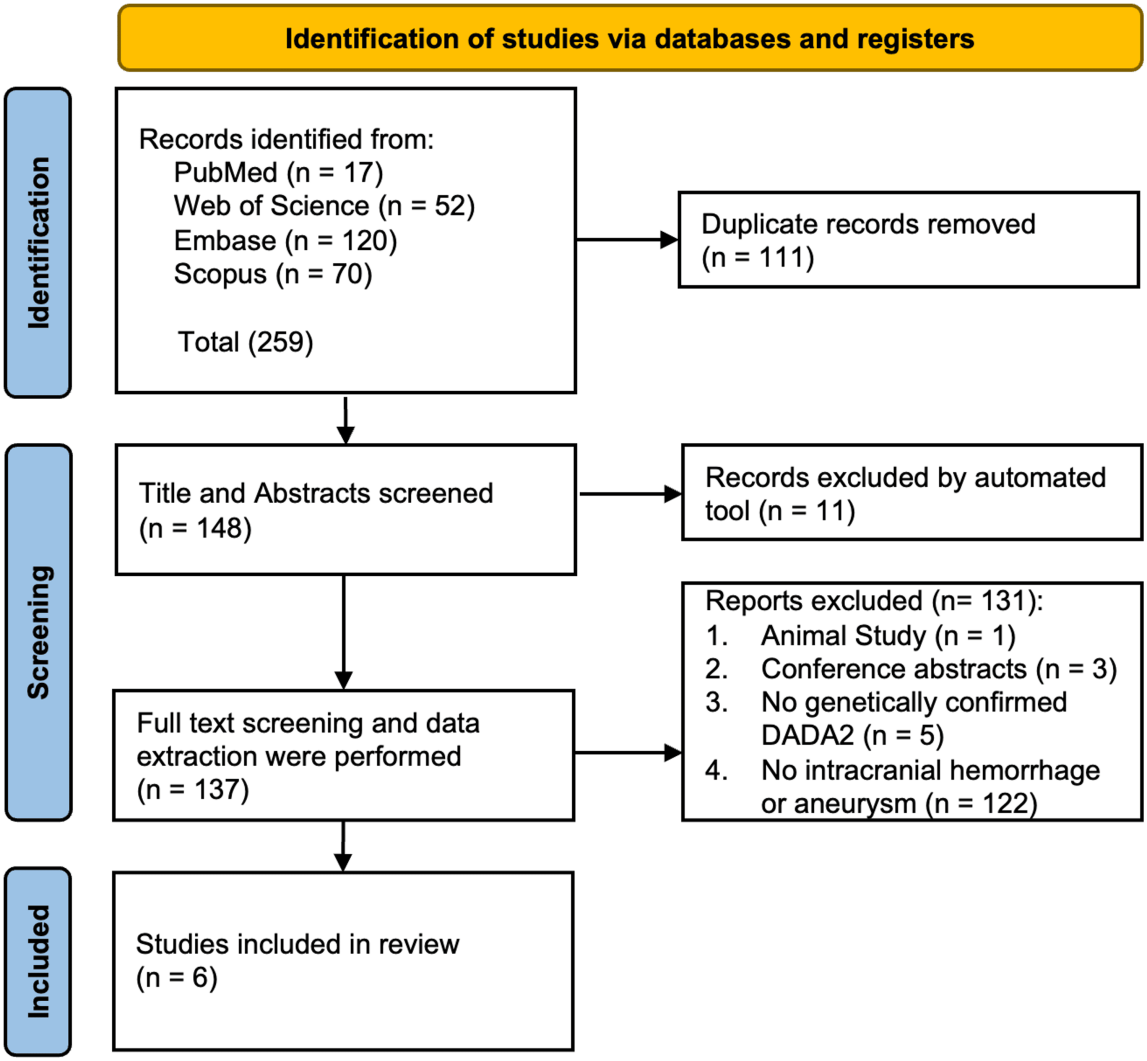
Preferred Reporting Items for Systematic Reviews and Meta-Analyses (PRISMA) flow diagram of the literature search and study selection process. A total of 259 records were identified through database searching (PubMed n = 17, Web of Science n = 52, Embase n = 120, Scopus n = 70). After removal of 111 duplicate records, 148 records remained for screening. Eleven records were excluded using an automated screening tool. The remaining 137 reports were retrieved and assessed for eligibility. Of these, 131 reports were excluded after full-text review due to an animal study (n = 1), conference abstracts (n = 3), the absence of genetically confirmed DADA2 (n = 5) or lack of intracranial hemorrhage or aneurysm (n = 122). Six studies were ultimately included in the review. Adapted from [10].

To identify all relevant manuscripts or case series, four major databases, PubMed, Web of Science, Embase, and Scopus, were systematically searched on January 17, 2026 (Figure 5). The search strategy combined keywords and subject headings related to (“ADA2” OR “DADA2”) AND (“aneurysm*” OR “hemorrhage”). Records identified through the database searches were imported into the Autolit platform (Nested Knowledge, St. Paul, MN, USA), which was used for duplicate removal as well as title and abstract screening. 

Reports were included if they described patients with genetically confirmed DADA2 and documented intracranial aneurysms on neurovascular imaging. Only peer-reviewed publications were considered. Animal studies, conference abstracts, and reports without genetically confirmed DADA2 or without documented intracranial aneurysms were excluded.

Full text screening and data extraction were performed by a single investigator, and relevant variables were recorded in a data table. For each case, age, sex, age at symptom onset, age at diagnosis, clinical presentation, hemorrhagic phenotype, aneurysm location, and treatment were extracted and are summarized in Table 1.

**Table 1:** Summary of reported DADA2 cases with cerebrovascular involvement and intracranial aneurysms Abbreviations: HTN, hypertension; SNHL, sensorineural hearing loss; VH, ventricular hemorrhage; aSAH, aneurysmal subarachnoid hemorrhage; ICA, internal carotid artery; VA, vertebral artery; MCA, middle cerebral artery; ACA, anterior cerebral artery; SCA, superior cerebellar artery; ACom, anterior communicating artery; TNF-α inhibitor, tumor necrosis factor alpha inhibitor; GI, gastrointestinal; IL-1 inhibitor, interleukin-1 inhibitor. Age is reported in years unless otherwise specified. When the exact ages at diagnosis or symptom onset were not reported in the original publication, this is indicated as not available (NA). Intracranial aneurysms were reported in all cases included in this table; however, in some reports, the precise anatomical location of the aneurysms was not specified. Hemorrhagic phenotype and aneurysm characteristics were reported as described in the original studies. Across reported cases, intracranial aneurysms in DADA2 are typically multiple and frequently involve atypical vascular territories, with a predominance in the posterior circulation and small- to medium-sized vessels, contrasting with the pattern observed in sporadic intracranial aneurysms.

Author and year	Age / Sex	Age of symptom onset	Age at diagnosis	Clinical presentation	Hemorrhagic phenotype	Location of aneurysm	Treatment
Navon et al. 2014 [1]	2 mo/F	2 mo	7 mo	Fever; digital necrosis; GI symptoms	VH	Yes (unspecified)	Glucocorticoids; cyclophosphamide
	29/M	7 mo	16	Fever; livedo reticularis; skin nodules; recurrent stroke; HTN	-	Yes (unspecified)	Glucocorticoids; cyclophosphamide; methotrexate; TNF-α inhibitor
Geraldo et al., 2021 [7]	18/F	5	17	Livedo reticularis; skin ulcers; arthralgia; recurrent strokes	aSAH	ACom; SCA	TNF-α inhibitor
Verschoof et al., 2023 [11]	29/M	27	NA	Ischemic stroke	aSAH	ICA	Aneurysmal clipping; TNF-α inhibitor
Agajany et al., 2023 [9]	42/F	Since childhood	42	Limb ulcers; livedo reticularis; SNHL; amaurosis fugax	-	MCA; ACA	TNF-α inhibitor
Saravanan et al., 2025 [12]	19/F	Since childhood	19	Recurrent ischemic strokes; cranial nerve palsies; anemia	-	VA	TNF-α inhibitor
Reynolds et al., 2025 [13]	17/F	8	17	Fever; livedo reticularis; oedema	aSAH, Recurrent SAH after 3 months	Basilar perforator (suspected)	Glucocorticoids; azathioprine; mycophenolate; TNF-α inhibitor
Present study	9/M	8	8	Hypertension; GI symptoms; Raynaud; peroneal palsy; ischemic stroke	aSAH	Multiple (posterior circulation; anterior spinal artery)	Colchicine; IL-1 inhibitor; TNF-α inhibitor

Results

In addition to our case, the literature review identified seven patients with genetically confirmed DADA2 and reported intracranial aneurysm formation (Table 1), resulting in a total of eight analyzed patients. Hemorrhagic cerebrovascular events were observed in five of the eight patients, including subarachnoid hemorrhage. Notably, four of the eight patients had confirmed aneurysmal subarachnoid hemorrhage, whereas one additional patient presented with ventricular hemorrhage. In contrast, three patients presented exclusively with ischemic manifestations. Aneurysms were described as multiple in four of the eight patients and were most frequently located in the posterior circulation or distal intracranial vessels. Specifically, posterior circulation involvement was reported in five of the eight patients, whereas anterior circulation aneurysms were specified in two of the eight patients. Spinal artery involvement was documented in one patient. Anti-TNF-α therapy was administered in seven of the eight patients, while surgical or endovascular aneurysm treatment was reported in one case. These findings suggest a predilection for distal and posterior vascular territories in DADA2-associated aneurysmal disease.

Discussion

While the classic triad of DADA2 includes early onset stroke, livedo racemosa, and hypogammaglobulinemia, our case emphasizes a less common but life-threatening neurovascular phenotype: the formation of multiple atypically located intracranial and spinal aneurysms leading to SAH [1,5,7].

Pediatric aSAH is rare and often associated with vascular malformations or underlying genetic vasculopathies, rather than the acquired cardiovascular risk factors more frequently seen in adults [14]. In this case, the diagnosis of DADA2 provided the clinical context, but the identification of the "culprit" lesion required high-resolution multimodal imaging. The presence of aneurysms in the posterior circulation and anterior spinal artery is consistent with inflammatory arteriopathy affecting small- and medium-sized vessels [7]. This distribution is in line with current pathophysiological concepts of DADA2, in which monocyte/macrophage-driven inflammation and impaired endothelial integrity contribute to structural vessel wall damage and fragility, predisposing to the formation of small, peripherally located aneurysms [5,8]. DSA remains the diagnostic gold standard in this clinical context, offering the superior spatial resolution required to identify small peripheral aneurysms that may remain radiographically occult on standard MRA or CTA [4,11], a diagnostic challenge exemplified by the present case.

However, the optimal management of DADA2-associated aneurysms remains unclear. The peripheral location, eloquent location, and presumed inflammatory fragility of the vessel walls render both surgical and endovascular approaches risky. Furthermore, the fragility of inflamed vessel walls in DADA2 increases the risk of iatrogenic rupture or dissection during endovascular navigation. Given these constraints, multidisciplinary management often shifts toward aggressive medical management. TNF-α inhibitors (e.g., etanercept and adalimumab) have emerged as the cornerstone of DADA2 therapy, effectively reducing the frequency of ischemic stroke and potentially stabilizing inflammatory vascular lesions [15,16]. However, their role in preventing the rupture of pre-existing aneurysms remains unclear.

According to the literature, central nervous system involvement represents a dominant disease manifestation, highlighting the vulnerability of the cerebral vessels to inflammatory injury. Hemorrhagic events generally occur in the background of prior neurological involvement and are frequently preceded by ischemic manifestations [11,13]. In the present patient, an ischemic episode several months before the aSAH supports the concept of stepwise progression from ischemic to hemorrhagic cerebrovascular involvement, potentially driven by cumulative vessel wall damage and increasing fragility [5,8]. Although the reported ages at onset range from childhood to adulthood, the pediatric presentation in our patient underscores that severe neurovascular complications may occur early in life.

In contrast to the solitary and surgically accessible aneurysms described in isolated reports, the patient presented with a diffuse aneurysmal phenotype with multiple lesions affecting the eloquent vascular territories. Comparable aneurysm configurations have previously been reported in pediatric patients diagnosed with polyarteritis nodosa (PAN), in whom similar distal posterior circulation aneurysms were observed; however, these reports predate the recognition of DADA2 and therefore lack genetic characterization [6,17]. In the present case, this morphology had direct therapeutic implications as both surgical and endovascular approaches carry a high risk of vessel injury. This pattern is consistent with the findings of our literature review, in which aneurysms were predominantly multiple, distally located, and frequently involved the posterior circulation. However, the additional involvement of the anterior spinal artery in our patient represents a particularly rare manifestation, further highlighting the extent of the underlying inflammatory arteriopathy.

This study has several limitations inherent to its single-case design and the accompanying literature review. First, conclusions regarding the natural history of aneurysm formation and rupture cannot be generalized beyond the individual cases described, and causal inferences linking inflammatory activity, aneurysm morphology, and hemorrhagic risk remain speculative. Second, the literature review was conducted by a single investigator, introducing potential selection bias. In addition, formal assessment of publication bias and inter-study heterogeneity was not feasible, and the small number of identified cases (n = 8) further limits the robustness and generalizability of the findings.

Second, although comprehensive imaging was performed, histopathological confirmation of aneurysm wall pathology was not available. As a result, assumptions regarding inflammatory vessel wall fragility are based on indirect imaging features and pathological concepts described in the literature.

Finally, the impact of immunomodulatory therapy on aneurysm stability could not be systematically assessed, as treatment decisions were guided by clinical necessity rather than by predefined protocols.

## Conclusions

This case highlights intracranial aneurysm formation as a rare, but clinically severe, neurovascular manifestation of DADA2. Therefore, DADA2 should be considered as an important differential diagnosis in pediatric patients presenting with SAH, particularly in the presence of multiple small, atypically located aneurysms. DSA remains the reference standard for comprehensive vascular assessment and may be required to delineate the full extent of vascular involvement. Early recognition is important, given that anti-TNF-α therapy has been associated with reduced cerebrovascular event recurrence. Given the multisystem nature of DADA2, optimal patient care requires close, multidisciplinary collaboration.
